# Stereochemistry and Charged State Influence Effector Outcomes of d‑2-Hydroxyglutarate Dehydrogenase Ligands

**DOI:** 10.1021/acs.biochem.5c00408

**Published:** 2025-09-08

**Authors:** Joanna Afokai Quaye, Giovanni Gadda

**Affiliations:** † Department of Chemistry, 1373 Georgia State University, Atlanta, Georgia 30302-3965, United States; ‡ Department of Biology, Georgia State University, Atlanta, Georgia 30302-3965, United States; § The Center for Diagnostics and Therapeutics, Georgia State University, Atlanta, Georgia 30302-3965, United States

## Abstract

d-2-Hydroxyglutarate dehydrogenase (D2HGDH) has recently received considerable attention due to the involvement of d-2-hydroxyglutarate in various medical conditions. This enzyme has been reported to diverge in substrate scope depending on whether its source is prokaryotic or eukaryotic. The D2HGDH from *Pseudomonas aeruginosa*, *Pa*D2HGDH, is of particular interest due to its requirement for *P. aeruginosa* survival via the l-serine biosynthesis pathway and its potential use as a therapeutic target against the bacterium. The enzyme, which is active on d-2-hydroxyglutarate (D2HG) and d-malate, is a Zn^2+^- and FAD-dependent dehydrogenase that employs metal-triggered flavin reduction in its catalytic mechanism. While *Pa*D2HGDH is the most extensively studied D2HGDH homologue, no studies have investigated the ligand-binding modalities in the enzyme, and—for that matter—any D2HGDH homologue. This study investigated the inhibition profiles of *Pa*D2HGDH by various D2HG and d-malate analogues. The study demonstrates that stereochemistry and functional groups at the C2 position of ligands are key determinants of binding to *Pa*D2HGDH. The enzyme recognizes d-isomeric ligands as substrates, with l-isomers acting as reversible inhibitors. Ligand binding requires bidentate coordination with the active site Zn^2+^ cofactor, with longer chain ligands and polar ligands having lower *K*_is_ and Δ*G* ^o^ values due to enhanced interactions with the highly polar active site. Hydrophobic and van der Waals interactions also contribute to ligand binding in *Pa*D2HGDH. The study concludes that *Pa*D2HGDH can be reversibly inhibited, providing a foundation for biochemical studies on *Pa*D2HGDH inhibitors, with direct applications to D2HG biosensor development.

## Graphical abstract

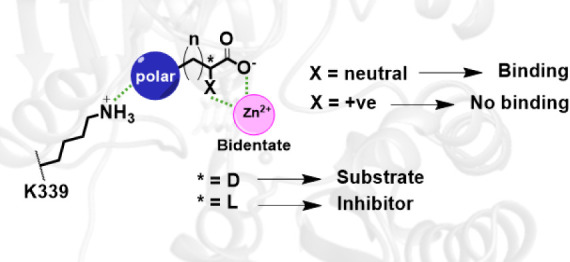

## Introduction

d-2-Hydroxyglutarate dehydrogenase (D2HGDH) (EC: 1.1.99.39; Uniprot ID: Q9I6H4) is an enzyme that has recently received considerable attention due to the involvement of its physiological substrate, d-2-hydroxyglutarate (D2HG), in several cancers and the neurometabolic disorder D2HG aciduria.^1–11^ Thus, D2HGHG homologues have recently emerged as biological markers in biosensors for various medical applications.^12–14^ D2HGDHs have been reported as metalloflavoproteins, although various D2HGDH homologues are active with varied metal cofactors.^15^ In a recent review on D2HGDH homologues from various species, the prokaryotic and eukaryotic enzymes have been reported to diverge from two distinct ancestors and appear to have differentially evolved to specialize in their functionality and α-hydroxy acid substrate scopes.^15^ While D2HG and d-malate were reported as substrates for D2HGDH homologues from prokaryotic and eukaryotic sources, d-lactate appeared primarily associated with the eukaryotic enzymes.^15^ Considering that the active site topology of D2HGDH homologues is conserved across all enzymes, it is important to understand why various D2HGDH homologues exhibit varied substrate reactivities, with some showing strict reactivity for D2HG only.^15^ While the substrate differences can be attributed to possible enzyme-specific structural motions that favor binding of specific substrates over others, no studies have been performed to elucidate the substrate-binding modalities in any D2HGDH homologue.

The most characterized D2HGDH homologue is *Pa*D2HGDH^15–21^ from *P. aeruginosa*, a multidrug-resistant pathogen known to cause human nosocomial infections.^22–25^ A recent D2HGDH gene knockout study in *P. aeruginosa* identified the enzyme as important for *P. aeruginosa* survival.^26,27^ *P. aeruginosa* depends on l-serine as a central intermediate for several biological processes, and *Pa*D2HGDH has been reported to play an essential role in *P. aeruginosa* l-serine biosynthesis.^26,27^ By converting D2HG to 2-ketoglutarate, *Pa*D2HGDH replenishes cellular levels of 2-ketoglutarate to drive *P. aeruginosa* l-serine biosynthesis, which is required for survival, making *Pa*D2HGDH a potential target for therapeutics against *P. aeruginosa*.^27^ *Pa*D2HGDH is a Zn^2+^ and FAD-dependent dehydrogenase that catalyzes the oxidation of the D2HG substrate to 2-ketoglutarate (Scheme 1).^19^ The enzyme is also active with d-malate as an alternative substrate, converting it to oxaloacetate (Scheme 1).^18^ Despite the absence of a crystal structure for the *Pa*D2HGDH enzyme, several *Pa*D2HGDH homology models have been reported for *Pa*D2HGDH.^15,18^ These models, which were built using various online protein prediction tools, such as AlphaFold3 ^15^ and SWISS-MODEL,^18^ share identical structural characteristics with the published crystal structure of the human D2HGDH homologue.^11,28^ The enzymes have fully conserved FAD-binding and substrate-binding domains, with the FAD-binding domain demonstrating a classical PCMH-type fold with two subdomains.^15,28^ Sequence alignment of various D2HGDH homologues revealed 36 fully conserved amino acid residues, with 18 found in the FAD-binding domain. The FAD-binding domain also contains a buried pyrophosphate-binding loop (PP-loop) characterized by an X_6_ **GG**X**T**X_11_ loop–β-strand–loop motif.^15^ The remaining 18 conserved amino acids are located in the substrate-binding domain, four of which are in the active site.^15^

**Scheme 1:**
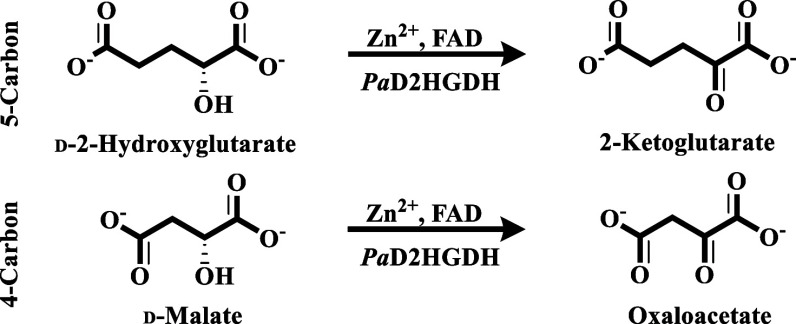
Catalytic Scheme of d-2-Hydroxyglutarate Dehydrogenase from *P. aeruginosa*

The fully conserved active site ligands of *Pa*D2HGDH have been reported to be H374, H381, E420, and H421, with H374, H381, and E420 serving as metal ligands for catalysis. The enzyme, which is reported to be Zn^2+^-dependent, is also active with Co^2+^, Ni^2+^, Mn^2+^, and Cd^2+^ as alternative metal cofactors for substrate oxidation.^19,21^ The *Pa*D2HGDH Zn^2+^ cofactor orients, binds, and polarizes the α-hydroxy acid substrate, leading to the loss of the C2–OH proton, which triggers a hydride transfer to the N5 atom of the flavin cofactor during substrate oxidation.^17,19,20^ Additionally, the enzyme has an optimum pH of ∼7.5 and requires an active site proton acceptor, which has a p*K*_a_ value of 8.3, for flavin reduction.^17,20^ Moreover, the enzyme is reported to have a partially conserved lysine residue, K339, which together with the Zn^2+^ cofactor has been proposed to be important for substrate binding in *Pa*D2HGDH.^15^ However, no studies have explored the ligand properties required to bind *Pa*D2HGDH or other D2HGDH homologues.

The present study used steady-state kinetic inhibition assays to investigate D2HG and d-malate analogs as inhibitors of recombinantly expressed His-tagged *Pa*D2HGDH. The study explores the structural moieties required for ligand recognition to yield various effector outcomes in *Pa*D2HGDH. This study is an initial step toward biochemical research focused on developing inhibitors targeting *Pa*D2HGDH, either for biosensor development through competitive binding assays or therapeutic design.

## Materials and Methods

### Materials

The *Pa*D2HGDH pET20b­(+) plasmid harboring a *C*-terminal 6x-His-tagged-PA0317 gene was purchased from GenScript (Piscataway, NJ) following in-lab plasmid design. The plasmid was sequenced via Psomagen USA to verify the presence of the wild-type gene. *Escherichia coli* strain Rosetta­(DE3)­pLysS was from Novagen (Madison, WI). BSA was purchased from Promega (Madison, WI). Luria–Bertani agar, Luria–Bertani broth, chloramphenicol, IPTG, lysozyme, phenazine methosulfate (PMS), PMSF, d/l-isocitric acid, d/l-3,4-dihydroxymandelic acid, and l-2-hydroxyglutarate were obtained from Sigma-Aldrich (St. Louis, MO). Ampicillin was purchased from ICN Biomedicals (Aurora, OH). d-Malic acid, d-mandelic acid, d/l-4-hydroxymandelic acid, tartronic acid, d-lactic acid, 2-ketoglutaric acid, d-aspartate, d-glutamine, and d-arginine were purchased from Alfa Aesar (Haverhill, MA). Oxalacetic acid was purchased from TCI Co. Ltd (Portland, OR). All other reagents were purchased at high purity.

### Expression and Purification of *Pa*D2HGDH

To obtain pure His-tagged *Pa*D2HGDH for kinetic studies, a 10 mL Luria–Bertani broth was supplemented with 100 μg/mL ampicillin and 34 μg/mL chloramphenicol and inoculated with frozen stocks of *E. coli* cells Rosetta­(DE3)­pLysS harboring the 6x-His *Pa*D2HGDH pET 20b­(+) plasmid. The cell cultures were used to inoculate 1 L of LB broth and incubated on a rotary plate at 37 °C and 180 rpm for 18 h. When the cell density reached an OD_600_ nm of ∼0.4, the temperature of the culture was then lowered to 18 °C while shaking on a rotary plate at 180 rpm. Protein expression was induced with 100 μM IPTG when the cell density reached an OD_600_ nm of ∼0.6–0.8. After ∼20 h of expression, the cells were harvested by centrifugation for 30 min at 2800*g* and 4 °C.

A lysis buffer containing 1 mM PMSF, 2 μg/mL DNase or RNase, 4 mg/mL lysozyme, 5 mM MgCl_2_, 300 mM NaCl, 10 mM imidazole, 10% glycerol, 1 mM ZnCl_2_, and 20 mM NaPO_4_, pH 7.4 was used to resuspend the wet cell paste in a ratio of 1 g of wet cell paste to 4 mL of lysis buffer. The suspended cells were then incubated for 30 min on ice while stirring. The resulting slurry was sonicated in 5 cycles, each comprising a 5-min run time with a 5-min off interval between cycles. Following sonication, the cell debris was removed by centrifugation at 11 200*g* for 30 min. To obtain the Zn^2+^-bound enzyme, the supernatant (cell-free extract) was purified to homogeneity using a Ni-NTA column equilibrated with 20 mM NaPO_4_, 10 mM imidazole, 300 mM NaCl, 1 mM ZnCl_2_, and 10% glycerol, pH 7.4 (Buffer A). The purification was carried out using a Unicorn AKTA Start purification system. Elution of the bound protein was achieved through a gradient from 0 to 100% 20 mM NaPO_4_, 500 mM imidazole, 300 mM NaCl, 1 mM ZnCl_2_, and 10% glycerol, pH 7.4 (Buffer B), with *Pa*D2HGDH eluting at ∼40% Buffer B. The solution containing the purified protein, typically 15 mL, was dialyzed against five 2-L changes of 10% glycerol, 20 mM NaPO_4_, 1 mM ZnCl_2_, pH 7.4, for 2 h each, at 4 °C, following an initial overnight dialysis (18 h) of the purified protein in 2-L of the same buffer. The purified *Pa*D2HGDH enzyme was stored in single-use aliquots in 10% glycerol, 25 mM NaPO_4_, pH 7.4, at −20 °C, and was stable for at least 6 months.

### Enzyme Activity and Inhibition Studies

The activity of *Pa*D2HGDH with various ligands was investigated by monitoring the initial rates of PMS-driven oxygen consumption with a computer-interfaced Oxy-32 oxygen monitoring system (Hansatech Instruments Ltd.). For each ligand assay, 15 to 60 mM of the ligand was tested in a reaction containing 0.48 mM enzyme, with a fixed 1 mM PMS concentration as the electron acceptor, in air-saturated 25 mM NaPO4 buffer, pH 7.4, at 25 °C.

To investigate the inhibition effects of the various ligands on *Pa*D2HGDH activity, inhibition assays were carried out with varying concentrations of both the inhibitor and d-malate as a substrate, while PMS was fixed at 1 mM as an artificial electron acceptor. Except for the oxaloacetate assay, for which d-malate concentration ranged from 1 to 60 mM, the d-malate concentration range was 1–40 mM for all other ligand assays. 2-Ketoglutarate ranged from 1 to 25 mM, oxaloacetate ranged from 0.5 to 15 mM, l-2-hydroxyglutarate ranged from 1.5 to 10 mM, l-malate ranged from 2 to 30 mM, d,l-isocitrate ranged from 1 to 20 mM, d-mandelate ranged from 5 to 50 mM, d,l-4-hydroxymandelate ranged from 0.5 to 7 mM, tartronate ranged from 4 to 60 mM, d-lactate ranged from 1 to 40 mM, d-aspartate ranged from 1 to 20 mM, d-glutamate ranged from 1 to 20 mM, and d-arginine ranged from 1 to 20 mM. The reaction conditions included air-saturated 25 mM NaPO_4_, pH 7.4, and 25 °C. For each ligand assay, a 0 mM ligand data point was collected for each d-malate concentration tested to obtain a control *Pa*D2HGDH apparent steady-state kinetic plot with d-malate. Enzymatic rates were expressed per active site oxidized flavin content. d-Malate was used since it yields faster turnover rates with *Pa*D2HGDH compared to the physiological substrate D2HG.

### Data Analysis

Data were analyzed using KaleidaGraph (Synergy Software, Reading, PA) or Enzfitter software (Biosoft, Cambridge, UK). For the apparent steady-state kinetic plots, the Michaelis–Menten equation was used. For the inhibition plots, the best fit of the initial rates of the PMS-driven oxygen consumption was obtained with eqs 1 or 2, which describe a competitive or noncompetitive inhibition pattern, respectively. In these equations, *k*_cat_ is the turnover number of the enzyme (*e*), *v*_*o*_ is the initial velocity of the reaction, *I* is the inhibitor concentration, *K*_is_ is the inhibition constant for inhibitor binding to free E and is given by the slope term, *K*_ii_ is the inhibition constant for inhibitor binding to the ES complex and is given by the intercept term, and *K*_a_ is the Michaelis constant for the d-malate substate.

1 $$\frac{v_{o}}{e}=\frac{k_{\mathrm{cat}}A}{K_{a}\left[1+\left(\frac{I}{K_{\mathrm{is}}}\right)\right]+A}$$

2 $$\frac{v_{o}}{e}=\frac{k_{\mathrm{cat}}A}{K_{a}\left[1+\left(\frac{I}{K_{\mathrm{is}}}\right)\right]+A\left[1+\left(\frac{I}{K_{\mathrm{ii}}}\right)\right]}$$

### Ligand Docking into *Pa*D2HGDH

The molecular docking of various *Pa*D2HGDH ligands to the enzyme was done using the Protenix Server^29^ and the SwissDock^30–32^ online protein prediction tools. To generate the Zn^2+^ and FAD-bound *Pa*D2HGDH structure, the *Pa*D2HGDH protein sequence obtained from the UniProtKB database was used along with the CCD code for the Zn^2+^ ion and the SMILES codes for FAD in the Protenix server. The resulting Zn^2+^ and FAD-bound *Pa*D2HGDH structure was visualized using UCSF Chimera.^33^ The target PDB file for *Pa*D2HGDH was generated by exporting the Protenix-generated *Pa*D2HGDH structure in the PDB format using UCSF Chimera.

Docking of various ligands was performed using the Protenix and SwissDock online tools for comparison. Initial docking data for all ligands were generated using the Protenix server by inputting the *Pa*D2HGDH sequence, the CCD code for Zn^2+^, and the SMILES codes for FAD and all ligands. Stereospecific binding of ligands to the *Pa*D2HGDH target was investigated using SwissDock by inputting the SMILES codes of the various ligands and the PDB file of the *Pa*D2HGDH target, which had Zn^2+^ and FAD already bound to the protein. The protein search box center was set at −1, −6, and 2 Å with search boxes sized 26, 24, and 23 Å. Cavity prioritization was set to buried with medium sampling exhaustivity, and the number of RICs was set to 1 for all ligands.

The predictions with the highest confidence from both servers, marked as cluster #0, were then visualized and analyzed using UCSF Chimera. To compare the binding orientations, geometries, and distances of the various C2 ligand variants of the 5-carbon-length D2HG substrate, the first member of each #0 cluster across all ligands generated by the SwissDock server, which can discriminate ligands based on stereochemistry, was analyzed using UCSF Chimera. However, the SwissDock server could not process positively charged amino acids. The SwissDock files for the 4-carbon-length d-malate and its C2 ligand variants were selected to match the same binding geometry and orientation as their 5-carbon counterparts in the UCSF Chimera analyses.

2D ligand contact maps of cluster #0 of all Protenix ligand docking complexes for the substrates, products, and d-amino acids were generated to predict ligand contacts and charge-state interactions with *Pa*D2HGDH using LigPlot+.^34,35^

## Results

### *Pa*D2HGDH Inhibition Assay

To investigate the ligand moieties required to bind *Pa*D2HGDH, structural variants of the 5-carbon-length D2HG and 4-carbon-length d-malate substrates were investigated under steady-state conditions at pH 7.4 and 25 °C. Each ligand was first tested as a substrate for *Pa*D2HGDH and was considered for inhibition studies if it showed no activity with the Zn^2+^ and FAD-bound *Pa*D2HGDH. For each inhibition assay, ligand and d-malate concentrations were varied at a fixed saturating PMS concentration, and the double reciprocal plots from the inhibition assays were considered.

The inhibition studies using the products of *Pa*D2HGDH substrate oxidation, 2-ketoglutarate for D2HG and oxaloacetate for d-malate, yielded enzyme inhibition as expected. Except for d/l-3,4-dihydroxymandelate, patterns with intersecting lines on the *y*-axis for all inhibitor concentrations in the double reciprocal plots were observed for all of the tested *Pa*D2HGDH ligands, as shown in the representative inhibition plot of the 2-ketoglutarate product of *Pa*D2HGDH oxidation of the substrate D2HG (Figure 1A). In agreement with the observed kinetic patterns, the best fits of the kinetic data were obtained with eq 1, which describes a competitive inhibition pattern as a function of the d-malate concentration. On the contrary, the d/l-3,4-dihydroxymandelate ligand demonstrated an inhibition pattern characterized by converging lines on the *x*-axis with an intersecting point in the negative quadrant of the *x*-axis at different inhibitor concentrations (Figure 1B). The d/l-3,4-dihydroxymandelate double reciprocal plot data were best fit with eq 2, which describes a noncompetitive inhibition pattern as a function of d-malate concentration. The kinetic parameters obtained from the various inhibition studies are summarized in Table 1. No inhibition of *Pa*D2HGDH was observed when d-aspartate, d-glutamate, and d-arginine were tested as ligands. For all inhibition assays, the Michaelis constant *K*_a_ for d-malate and the enzyme turnover number *k*_cat_ for the assay were ≤2-fold different from the published kinetic parameters of 8 mM and 24 s^–1^, respectively, for the enzyme.^15,17,19^ These differences were deemed insignificant and were not further investigated in this study.

**Figure 1:**
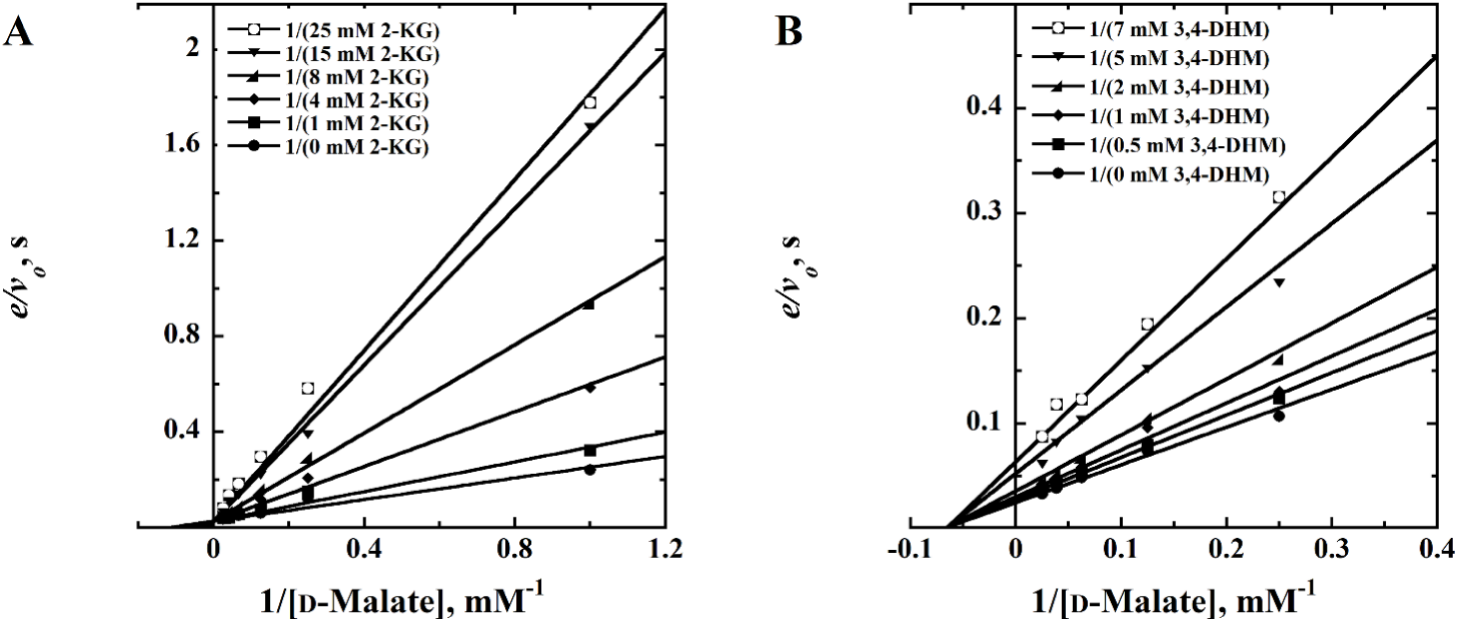
Inhibition of *Pa*D2HGDH with various ligands as inhibitors and d-malate as the substrate. Double reciprocal plots of *Pa*D2HGDH inhibition with (A) 2-ketoglutarate, showing patterns of competitive inhibition (eq 1), and (B), d/l-3,4-dihydroxymandelate, showing patterns of noncompetitive inhibition (eq 2). As reported in Table 1, all other inhibitors followed the same pattern as 2-ketoglutarate, yielding competitive inhibition of *Pa*D2HGDH. Concentrations of d-malate ranged from 1 to 60 mM. Assays were carried out in 25 mM NaPO4, pH 7.4, and 25 °C using a computer-interfaced Clark-type Oxy-32 oxygen monitoring system (Hansatech Instruments Ltd.) by following the PMS-driven consumption of air-saturated oxygen in reaction solutions as a reporter for enzyme activity.

**Table 1:**
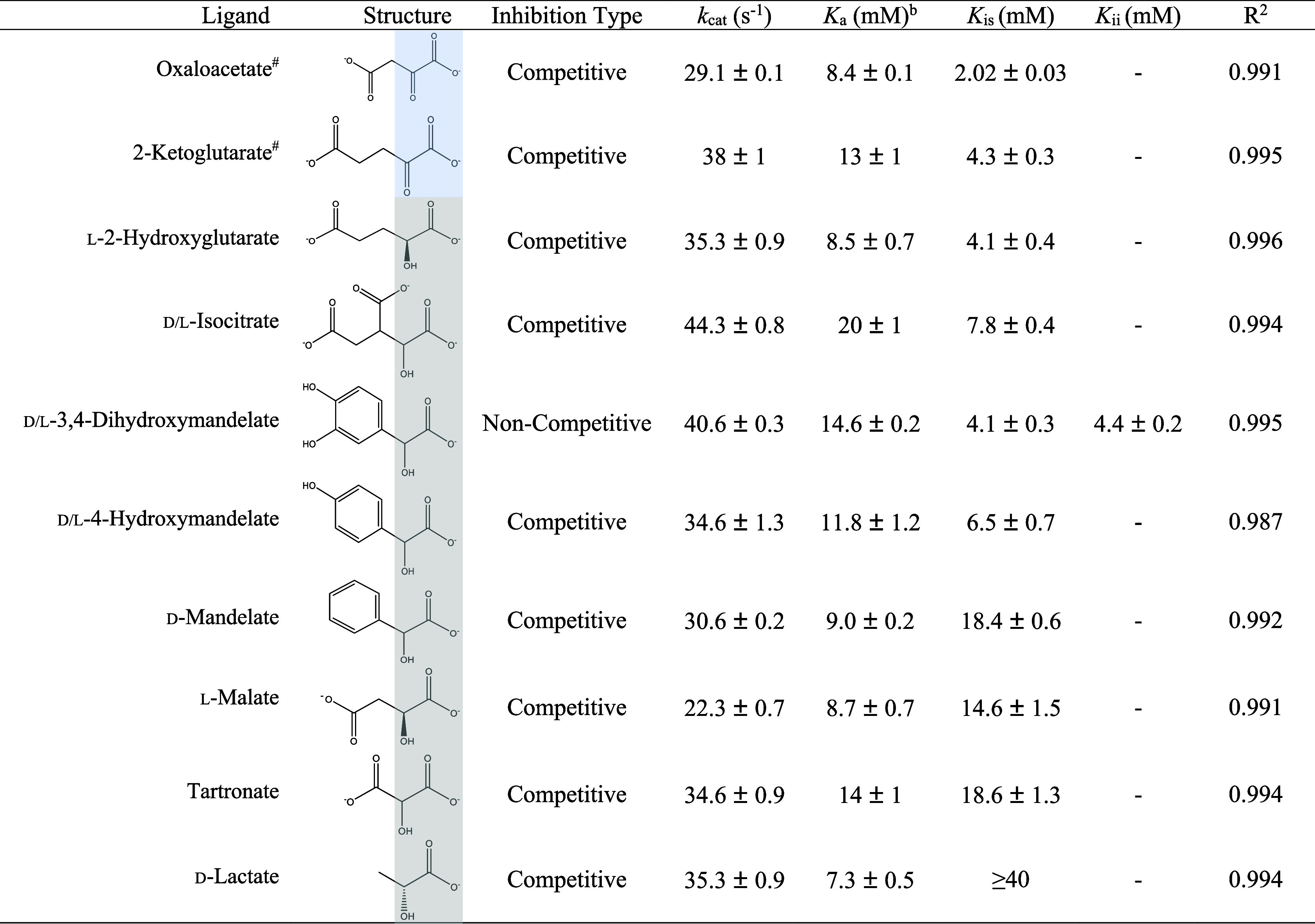
Steady-State Kinetic Parameters of *Pa*D2HGDH Inhibitors^a^ ^c^

a Activity assays were carried out in 25 mM NaPO_4_, pH 7.4, and 25 °C with an enzyme concentration ranging from 48 nM to 0.48 mM; d-malate concentrations ranged from 1 to 60 mM; and PMS concentration was fixed at 1 mM across all ligands tested.

b The *K*_a_ parameter values are the Michaelis constant values for d-malate in each inhibition assay.

c #Reaction products of *Pa*D2HGDH catalysis: 2-ketoglutarate for the d-2-hydroxyglutarate substrate and oxaloacetate for the d-malate substrate. No inhibition was observed for d-aspartate, d-glutamine, and d-arginine as ligands.

To investigate the energetic contribution of a methylene group to ligand binding in *Pa*D2HGDH, plots of the log *K*_is_ parameters and calculated Δ*G* ^o^ (Gibbs free energy for binding (Δ*G* ^o^ = *RT* ln (*K*_is_))) values against the aliphatic alpha-hydroxy acid inhibitor series were analyzed. The data included the *K*_is_ parameter and calculated Δ*G* ^o^ values obtained for l-2-hydroxyglutarate (2 CH_2_ groups), l-malate (1 CH_2_ group), and tartronate (0 CH_2_ groups). There was an observed dependence of the log *K*_is_ parameter and Δ*G* ^o^ value on the number of methylene groups in the inhibitor. With increasing inhibitory methylene groups, decreasing log *K*_is_ and Δ*G* ^o^ values were observed. A linear fit of the data yielded slopes of 0.33 ± 0.13 for the log *K*_is_ plot and 0.45 ± 0.18 for the Δ*G* ^o^ plot. The smallest *K*_is_ and lowest Δ*G* ^o^ values were observed for the inhibitor with the highest number of methylene groups, l-2-hydroxyglutarate (Figure 2).

**Figure 2:**
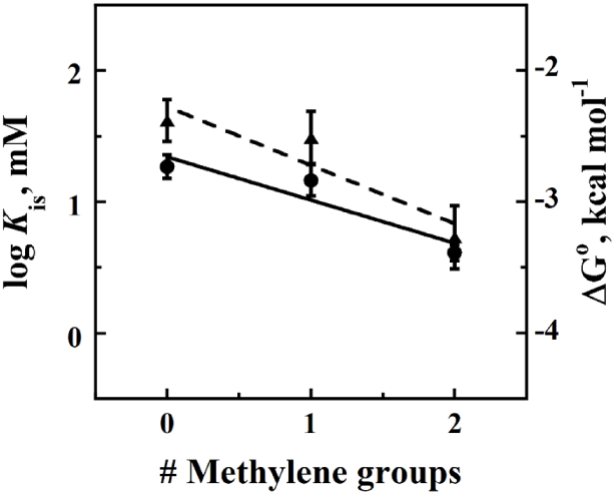
Dependence of the log *K*_is_ and Δ*G* ^o^ values on the number of methylene groups of aliphatic alpha-hydroxy acid *Pa*D2HGDH inhibitors. Log *K*_is_ values are shown as ●, while Δ*G* ^o^ values are shown as ▲. Assays were carried out in 25 mM NaPO4, pH 7.4, and 25 °C using a computer-interfaced Clark-type Oxy-32 oxygen monitoring system (Hansatech Instruments Ltd.) by following the PMS-driven consumption of air-saturated oxygen in reaction solutions as a reporter for enzyme activity. Concentrations of d-malate ranged from 1 to 60 mM. Initial rates of reaction were fit to eq 1. The line represents a linear fit of the data to *y* = −0.328*x* + 1.34 for the log *K*_is_ plot and *y* = −0.445*x* – 2.28 for the Δ*G* ^o^ plot. Data were computed from *K*_is_ parameter values obtained for l-2-hydroxyglutarate (2 CH_2_ groups), l-malate (1 CH_2_ group), and tartronate (0 CH_2_ groups).

### Ligand Docking into *Pa*D2HGDH

To investigate the binding geometries and orientations of the various *Pa*D2HGDH ligands, docking analyses were carried out using the Protenix-generated Zn^2+^ and FAD-bound *Pa*D2HGDH targets in the SwissDock online protein prediction tool. SwissDock was used to investigate the binding discrepancies between the C2-variants of the D2HG and d-malate substrates. The binding analyses included both *Pa*D2HGDH substrates as a control and tested their l-isomers, which have the same functional groups but differing spatial arrangements; their products, which have keto groups at C2 instead of hydroxyl groups; and their d-amino acid counterparts. Each docking analysis returned ∼200 docking positions for the various ligands.

For the 5-carbon-length D2HG series, the prediction with the highest confidence for each ligand, marked as cluster #0 and cluster member #1, the first docking prediction, was considered and analyzed using UCSF Chimera. D2HG and all ligands, except for the d-amino acid counterparts, docked in the active site cavity of *Pa*D2HGDH (Figure 3A–C), forming a bidentate interaction between the *Pa*D2HGDH Zn^2+^ cofactor, the ligand C1 carboxylate oxygen, and the C2 oxygen (hydroxy or keto acid) atoms. While the C2 hydrogen was pointed toward the flavin N5 atom in the docking files of the d-isomeric ligands, the C2 hydrogen atom was pointed away from the flavin N5 atom in the l-isomeric ligand. In all cases, the ligand tails interacted with an active site lysine residue (K339), and hydrogen bond interactions were observed between all ligands with C2 keto or hydroxyl groups and an active site histidine residue (H421). The SwissDock server could not predict docking positions for the d-amino acid counterparts having a positive charge on the amino group at the C2 position. When the neutral d-amino acid counterparts were docked instead, the data yielded results comparable to those of other hydroxy or keto ligands (data not shown).

**Figure 3:**
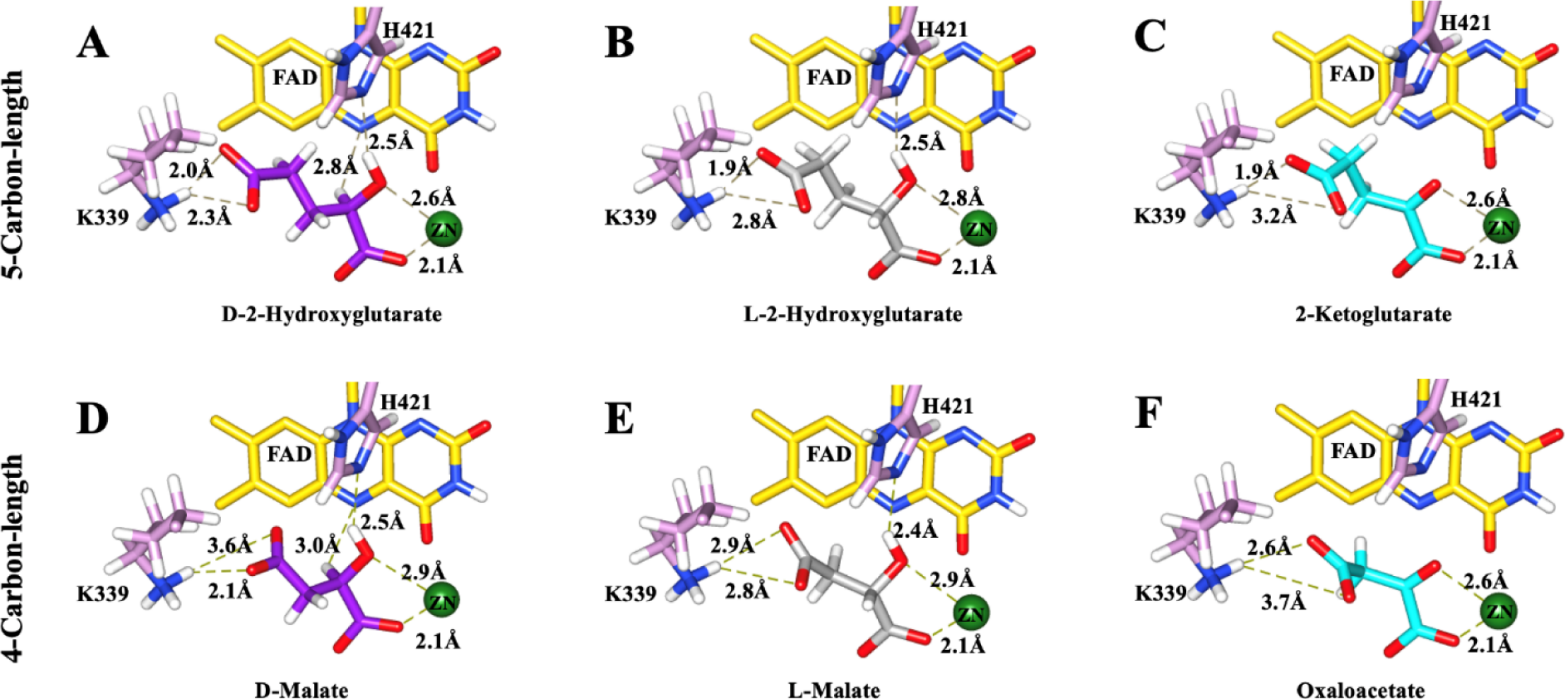
SwissDock^30–32^ predictions of *Pa*D2HGDH binding to D2HG, d-malate, and their C2-variants. A view of the active site interactions of *Pa*D2HGDH with (A) D2HG; (B) L2HG; (C) 2-ketoglutarate; (D) d-malate; (E): l-malate; and (F): oxaloacetate. The C2-hydrogen atoms in the l-isomeric substrate analogs point away, while those in the d-isomeric substrate analogs point toward the flavin N5 atom. *Pa*D2HGDH d-isomeric substrates are shown in purple, l-isomeric substrate analogs are shown in gray, and products are shown in cyan. The isoalloxazine ring of the FAD cofactor is shown in yellow; the Zn^2+^ cofactor is shown as a green sphere; the active site residues are shown in plum; nitrogen atoms are shown in blue; oxygen atoms are shown in red; and hydrogen atoms are shown in white. The protein structures were visualized using UCSF Chimera.^33^

For the 4-carbon-length d-malate series, docking predictions were selected to match the geometries and orientations of their 5-carbon-length counterparts in the D2HG series. The data yielded similar results for d-malate and its C2-variant ligands, as was observed for their 5-carbon-length counterparts (Figure 3D–F). All ligands, except for the positively charged d-amino acid counterparts, formed a bidentate interaction between the *Pa*D2HGDH Zn^2+^ cofactor and the ligand’s C1 carboxylate oxygen and C2 oxygen (hydroxy or keto acid), with interactions between the ligands and active site residues K339 and H421 and the flavin N5 atom where possible. When neutral forms of the d-amino acids were tested, the binding interactions were similar to those observed for the other hydroxy or keto ligands (data not shown).

### Ligand Contacts with *Pa*D2HGDH

Due to the inability of the amino acids to inhibit *Pa*D2HGDH activity with d-malate, the binding interactions of d-aspartate, d-glutamate, and d-arginine to *Pa*D2HGDH were interrogated using docking studies. Since SwissDock could not generate any docking outputs for the positively charged amino acids, docking analyses were carried out using the Protenix online protein prediction tool, with binding analyses of the *Pa*D2HGDH substrates and products run as controls. Additionally, docking analyses were performed for all *Pa*D2HGDH-confirmed inhibitors to establish their contact with the enzyme. Docking analyses for all *Pa*D2HGDH ligands yielded 5 docking positions for each ligand tested. The binding contacts of the first prediction for each ligand, having the highest confidence and marked as cluster no. 0, were then considered and visualized using LigPlot+.

When the *Pa*D2HGDH substrates and products were used, the data yielded ligand contacts similar to the SwissDock predictions, as expected (data not shown). Additionally, all α-hydroxy acid ligands followed binding patterns that mimicked the substrate binding behavior in *Pa*D2HGDH (data not shown). However, the ligand contacts for the amino acids were different. Typically, docking predictions took 5 min or less for all ligands tested to generate results. However, predictions for the positively charged d-amino acids took more than 12 h to generate results, with binding of ligands primarily away from the active site. The data suggest that the amino acids are less likely to bind *Pa*D2HGDH at pH 7.4, when their amino groups carry a +1 charge.

When neutral forms of the d-amino acids were docked, the predictions for d-aspartate and d-glutamate still deviated from the data obtained from the SwissDock docking results for the other hydroxy or keto acid counterparts. Additionally, unlike the SwissDock data obtained for the neutral amino acids, Protenix docking predictions of neutral forms of both d-aspartate and d-glutamate deviated from the typical C1–C2 bidentate interaction. Instead, their C1 carboxylate atoms interacted with the active site residue K339. Moreover, the Zn^2+^ cofactor made contact with the carboxylate tails and C2 amino groups of the uncharged amino acids (Figure 4).

**Figure 4:**
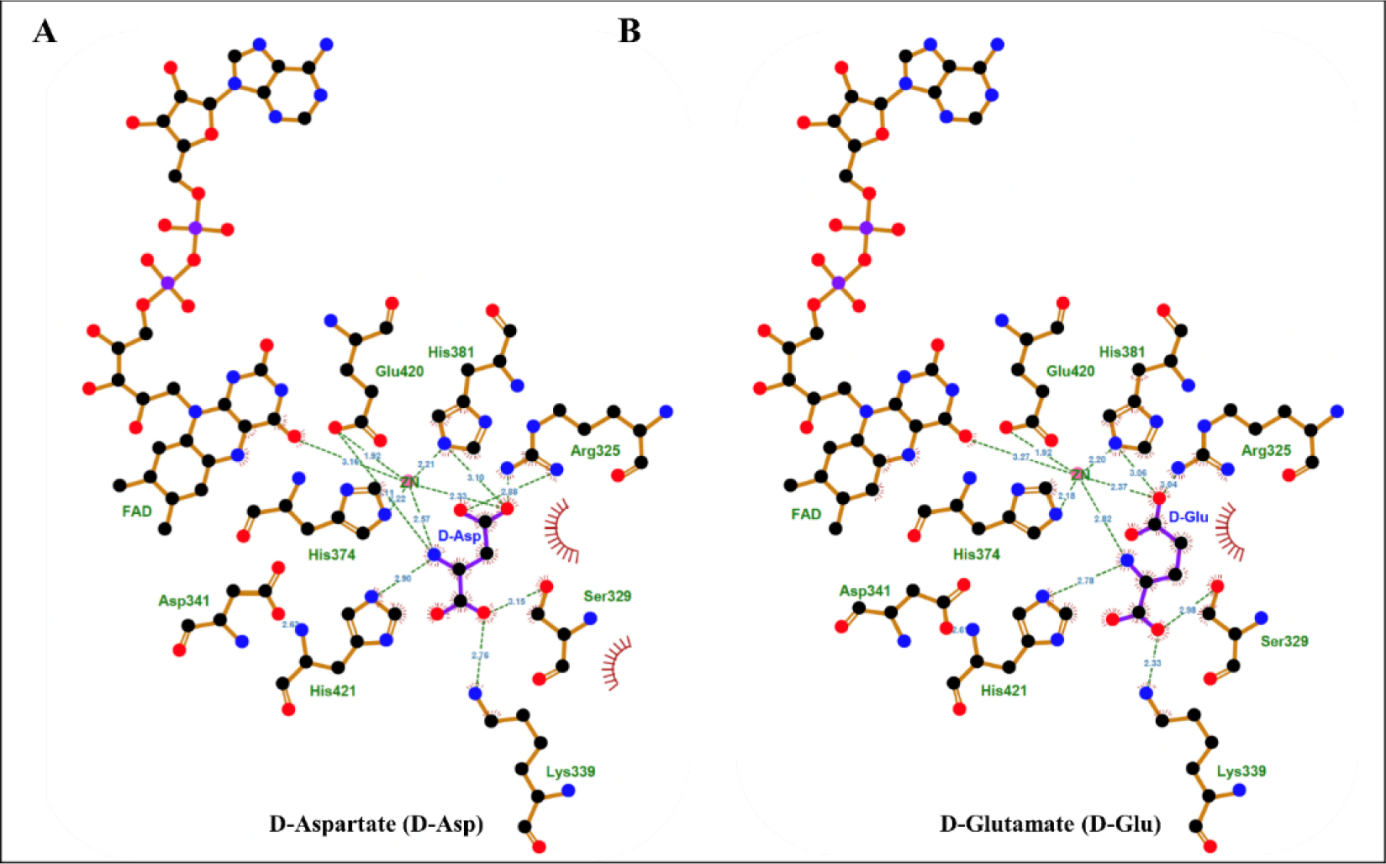
LigPlot+ ^34,35^ ligand contact maps of *Pa*D2HGDH binding to the neutral d-amino variants of the *Pa*D2HGDH substrate. A view of the active site interactions of *Pa*D2HGDH with (A) d-aspartate and (B) d-glutamate. The *Pa*D2HGDH target residues are shown with tan bonds, ligands with purple bonds, carbon atoms as black spheres, nitrogen atoms as blue spheres, and oxygen atoms as red spheres. Hydrophobic contacts are shown as crimson arches. Hydrogen bonds are shown as green dashed lines. The docking files were generated using the Protenix online tool.^29^

## Discussion

In the present study, D2HG and d-malate analogs have been tested as ligands and inhibitors of *Pa*D2HGDH to investigate the structural moieties required for *Pa*D2HGDH recognition of various ligands. Additionally, the study identified specific ligand properties responsible for various effector outcomes in *Pa*D2HGDH. This study is the first to investigate ligand modalities required for binding any D2HGDH homologue, as a first step toward understanding the varied substrate scopes across various D2HGDH homologues. Furthermore, the study represents a crucial step in understanding necessary ligand interactions in this class of enzymes for the selection and design of D2HGDH inhibitors that could be explored for biosensor development. Moreover, the *Pa*D2HGDH ligand-binding modalities could be explored for *Pa*D2HGDH-specific inhibitors for therapeutic design. The data demonstrate that the stereochemistry and functional group at the C2 position of various ligands are the main determinants for effector outcomes in *Pa*D2HGDH. Detailed analyses of the experimental data are discussed below.

### *Pa*D2HGDH Ligand Outcomes Depend on the Stereospecificity at the Ligand C2 Center

This conclusion is supported by the inhibitory effects of various *Pa*D2HGDH ligands (Table 1). Altering the stereochemistry at the C2 center of the *Pa*D2HGDH ligands from the d- in the D2HG and d-malate substrates to the l- in L2HG and l-malate drastically reversed their roles from being substrates to being inhibitors of *Pa*D2HGDH catalysis. The data demonstrate for the first time the strict stereoselectivity of *Pa*D2HGDH for its substrates. The lack of *Pa*D2HGDH reactivity with the l-isomeric ligands can be explained by the binding geometries of the different stereoisomeric ligands in the active site of the enzyme, as revealed by SwissDock ligand binding analyses (Figure 3). In the d-isomeric substrates, the C2 hydrogen atom points toward the flavin cofactor, positioning it at the right angle and distance from the flavin N-5 atom for a hydride transfer reaction required for enzyme catalysis.^17,20^ In contrast, when the stereochemistry is flipped in the l-isomeric ligands, the C2 hydrogen atom points away from the flavin cofactor, preventing any possible hydride transfer reactions for enzyme catalysis. Thus, although both stereoisomers bind to the metal and are activated for catalysis by the Zn^2+^ cofactor,^19,20^ the observed effects of the ligands are reversed due to the different binding geometries of the C2 substituents of the various ligands, which position the l-isomeric ligands in a catalytically incompetent geometry.

An alternative explanation for the observed reverse roles of the various *Pa*D2HGDH ligands upon switching their stereospecificity may be due to altered Zn^2+^ cofactor properties. Zinc typically adopts tetrahedral or distorted octahedral geometries in biological systems.^36^ A recent study on Zn^2+^_4_L_4_ cages demonstrates how ligand-induced chirality at the metal center itself could lead to enantioselective catalysis.^37^ The chirality at the *Pa*D2HGDH ligand’s C2 center can alter how the donor carboxylates or hydroxyls in the various ligands orient toward Zn^2+^, affecting the ligand field strength, metal–ligand bond angles, and electronic distribution between the ligands and the Zn^2+^ center.^38,39^ Consequently, the ability of the Zn^2+^ cofactor to activate the substrate or stabilize transition states may be altered, changing the outcome of the ligand from being a substrate in one stereoisomeric configuration to being an inhibitor in the other. Moreover, the l-isomers may introduce steric clashes or misalignments that prevent optimal Zn^2+^ coordination in *Pa*D2HGDH. Even if Zn^2+^ binds, the overall geometry may be catalytically incompetent, as seen with l-malate and L2HG in the current study. Hence, it is not just about binding but about productive binding of the ligand to the Zn^2+^ cofactor that leads to *Pa*D2HGDH turnover. Considering that the *Pa*D2GHDH Zn^2+^ cofactor functions to polarize and activate the α-hydroxy acid substrate and stabilize the carboxylate negative charge during catalysis,^15–21^ if the ligand’s stereochemistry positions the C1 carboxylate and C2 hydroxyl groups away from the Zn^2+^ cofactor, subsequent proton shuttling or electron transfers could be disrupted during catalysis, leading to altered catalytic outcomes of the Zn^2+^ in the presence of various ligand stereoisomers. This proton shuttling or electron transfer is especially relevant in *Pa*D2HGDH, where the C2 hydrogen must be aligned for hydride transfer to the flavin cofactor.^20^

### *Pa*D2HGDH Ligand Binding Requires Bidentate Ligand Coordination with the Metal Cofactor

Evidence supporting this conclusion comes from inhibition (Table 1) and docking analyses (Figures 3–4) of *Pa*D2HGDH with various ligands. Inhibition of *Pa*D2HGDH was observed only for ligands with either a hydroxyl or keto group at the C2 position. This observation can be explained as a bidentate interaction between the Zn^2+^ cofactor and the C1 carboxylate and C2 hydroxyl or keto oxygen groups, ensuring the ligands bind and are in a favorable position in the active site to elicit their effects on the enzyme.^17,19,20^ Considering the ligand structures, the bidentate Zn^2+^ cofactor binding explains why all *Pa*D2HGDH inhibitors, save for d/l-3,4-dihydroxymandelic acid, yielded competitive inhibition profiles since they compete with the substrate for the same binding site in the free enzyme, which is most likely the active site. The alternative ligand binding site for the negatively charged noncompetitive *Pa*D2HGDH inhibitor d/l-3,4-dihydroxymandelic acid, which binds to the enzyme–substrate (ES) complex species, is likely a nonspecific positively charged pocket outside the active site, since the substrate in the ES complex occupies the active site. Such nonspecific positively charged pockets could be on the protein surface, since *Pa*D2HGDH has been reported to have patches of positively charged surface pockets.^19^ Alternatively, the polar groups on the noncompetitive ligand could form hydrogen bond interactions with the backbone amino and carbonyl groups at various sites on the protein.

Another possible explanation for the noncompetitive inhibition observed for d/l-3,4-dihydroxymandelic acid is a likely chelation of the active site Zn^2+^ cofactor by the catechol moiety of the ligand. The Zn^2+^ chelation by d/l-3,4-dihydroxymandelic acid likely depletes or interferes with the *Pa*D2HGDH essential Zn^2+^ cofactor, rendering the enzyme incompetent, even if the substrate binds. Considering that the catechol binds to the Zn^2+^ cofactor and perturbs the enzyme functionality, rather than directly competing with the substrate, there is likely little to no protection against the chelation effect on *Pa*D2HGDH by the d-malate substrate. Thus, the inhibition profile appears noncompetitive, instead of the expected competitive inhibition for an α-hydroxy acid binding to *Pa*D2HGDH. This *Pa*D2HGDH inhibition through Zn^2+^ chelation was previously observed when the enzyme was treated with EDTA.^19^ Moreover, catechol inhibition of metalloenzymes via Zn^2+^ chelation has been previously reported for Zn^2+^-dependent metalloenzymes like thermolysin and human matrix metalloproteases.^40^

Given that d-glutamate and d-aspartate maintain the chain length and stereochemistry of D2HG and d-malate, respectively, with the only difference between the molecules being the replacement of a hydroxyl group in the substrates by an amino group in the amino acids, the amino acid binding geometries are expected to be similar to those of the other hydroxy and keto acid ligands (Figure 3). The lack of effect of the amino acids, including d-glutamate and d-aspartate, on *Pa*D2HGDH catalysis suggests that these amino acids do not bind to the enzyme since they did not show any activity as substrates. A likely explanation for the lack of binding of d-glutamate and d-aspartate to *Pa*D2HGDH is the presence of a positive charge on the amino group at pH 7.4, which results in the repulsion of the amino acid headgroup by the positively charged Zn^2+^ cofactor in the active site. Thus, the binding of these amino acid ligands is unfavored at pH 7.4, which was the working pH for the inhibition studies. Indeed, when the docking assays were repeated with neutral amino acid forms, binding was observed in *Pa*D2HGDH, as expected for a neutral amino headgroup interaction with the enzyme. Conversely, docking of positively charged amino acids yielded no meaningful results. Considering that both the SwissDock (Figure 3) and Protenix predictions (Figure 4) of the neutral d-amino acid binding interactions in *Pa*D2HGDH involve the amino headgroup coordination with the Zn^2+^ cofactor, successful binding of the amino acids to the enzyme is inconceivable at pH 7.4 when the amino group has a +1 charge, irrespective of the binding orientation following either docking prediction.

### *Pa*D2HGDH Can Be Reversibly Inhibited

Evidence supporting this conclusion comes from the inhibition profiles of *Pa*D2HGDH with various C2 variants of D2HG and d-malate (Table 1), apart from the d-amino acids. In all cases, the inhibition of *Pa*D2HGDH by the various ligands was reversible, either competitive (9 out of 10) or noncompetitive (1 out of 10), allowing for noncovalent ligand binding in the *Pa*D2HGDH active site. Except for oxaloacetate, which had a *K*_is_ value lower than the *K*_m_ value for d-malate, all other *Pa*D2HGDH inhibitors had *K*_is_ values that were similar to or above the reported 8 mM *K*_m_ value for d-malate,^17,18^ suggesting that, with minimal addition, d-malate can readily overcome the inhibitory effects of these ligands in *Pa*D2HGDH. Given that the *K*_m_ value for D2HG is 100-fold lower than that for d-malate in *Pa*D2HGDH,^17,18^ the ability of D2HG to alleviate any inhibition of the tested ligands in *Pa*D2HGDH will be much stronger than that of d-malate, requiring small amounts of D2HG to overturn *Pa*D2HGDH inhibition. These *K*_is_ values render these inhibitors even more attractive for competitive biosensor assays for D2HG detection by *Pa*D2HGDH^41–44^ by ensuring targeted displacement of active site ligands and reducing false negatives arising from nonspecific binding of target molecules to the enzyme.^45–47^ This competitive ligand binding mechanism not only addresses the existing issues of high cost and sophistication of D2HG detection methods^48–51^ but ensures the biosensor responds primarily to the presence of the target molecule when using enzymes, a promising avenue in the field. However, developing an effective and robust competitive binding assay will require further optimizations and strategies to overcome possible challenges such as the signal-to-noise ratio, reproducibility, and striking an equilibrium between the competing ligands in *Pa*D2HGDH. To this end, one avenue that could be explored for D2HG biosensor development using *Pa*D2HGDH is coupling the enzyme inhibitors or ligands to fluorescent probes that offer high sensitivity and selectivity while minimizing the signal-to-noise ratio of the detection method.

While this reversible inhibition may be excellent for biosensor development, it poses a challenge to therapeutic development, requiring nonreversible enzyme inhibition at minimal concentrations.^52,53^ Thus, studies on therapeutic agents that target *Pa*D2HGDH should explore ligand moieties that can trigger covalent adduct formation between the ligand and the enzyme to yield a dead-end enzyme complex during *Pa*D2HGDH catalysis.^53^ Such ligand moieties could include catechol groups for the metal cofactor chelation, as observed for d/l-3,4-dihydroxymandelic acid (*vide supra*), since the enzyme is known to be inactive without the metal cofactor.^19^ Notably, ligand binding in *Pa*D2HGDH is enhanced by the active site residue K339 (*vide infra*). This interaction, if explored, could be targeted for lysine-directed covalent chemistry to yield high-affinity irreversible enzyme inhibitors.^54^ Nonetheless, this study provides valuable information and a foundation for screening the pharmacokinetic properties of various ligands that could be explored as irreversible *Pa*D2HGDH inhibitors for therapeutic purposes.

### *Pa*D2HGDH Inhibition Is Enhanced by Increasing Ligand Chain Length

This conclusion is supported by the inhibition profiles of the aliphatic C2-hydroxyl inhibitor series having 0–2 methylene groups. There was an observed increase in the inhibition constant for the various aliphatic C2-hydroxyl inhibitors from the longest to the shortest inhibitor. The smallest *K*_is_ value, corresponding to the most potent inhibitor, was reported for L2HG, which has 2 methylene groups. In contrast, the largest *K*_is_ value, corresponding to the least potent inhibitor, was reported for tartronate, which has no methylene groups (Table 1 and Figure 2). The free energy contribution of a methylene group toward ligand binding in *Pa*D2HGDH, calculated from the slope of the plot of the log *K*_is_ value vs methylene group number, is estimated to be ∼0.45 kcal/mol. This independently calculated free energy contribution is comparable to the 0.45 kcal/mol slope of the plot of Δ*G* ^o^ versus methylene group number. The energetic contribution of the methylene group binding to *Pa*D2HGDH is comparable to ∼0.7 kcal/mol reported for transferring a methyl group from n-octanol to water.^55^ However, this methylene energetic contribution is relatively weaker than the ∼2.3 kcal/mol energetic contribution previously reported for a carboxylate tail interaction with *Pa*D2HGDH K339.^18^

While increasing the ligand tail length could be counterproductive due to steric clashes or excessive flexibility, the outcome of the increased ligand tail solely relies on the topology of the ligand binding site and its complementarity to the ligand. In a previous study on a racemase from *Mycobacterium tuberculosis*, increasing the chain length of inhibitors significantly increased the binding affinity of the ligand for the enzyme, with a reported methylene group contribution of 0.32 kcal/mol.^56^ In a similar study on carbonic anhydrase, ligand tail elongation was reported to increase desolvation of the greasy tails and the hydrophobic wall of the enzyme binding site, thereby favoring ligand binding to carbonic anhydrase with a methylene group free energy contribution of 0.37 kcal/mol.^57^ These reported free energy contributions are comparable to the observed methylene free energy contribution of ∼0.5 kcal/mol in this study. Moreover, in a previous study on choline oxidase, a similar decrease in inhibitor *K*_is_ values was observed with increasing methyl groups on the ammonium headgroup of choline.^58^ In that study, the trimethylammonium moiety was identified as critical for ligand binding in choline oxidase due to the hydrophobic nature of the choline oxidase active site, with one methyl group contributing ∼1 kcal/mol to the binding energy.^58^ In the case of *Pa*D2HGDH, the increased free energy of binding with an increased ligand tail length is likely due to an enhanced ligand interaction with the active site K339 residue upon ligand tail elongation.

Since all tested ligands have carboxylate tails, shortening the carbon chain length should impact the ligand interaction with the *Pa*D2HGDH K339 residue. The lack of a drastic drop in the energetic contribution, as expected from the loss of carboxylate interaction upon reducing the carbon chain length from L2HG to tartronate, can be explained by the likely flexibility of the *Pa*D2HGDH K339 side chain. The K339 flexibility allows it to move and bind ligands of varying chain lengths, resulting in interactions with ligand carboxylate tails despite the decreased chain length from L2HG to tartronate. Indeed, similar distances were observed for the interactions of K339 with the ligand tails when L2HG, l-malate, and tartronate were docked to *Pa*D2HGDH (Figure 5), suggesting similar carboxylate contributions across the various ligands. Hence, the different *K*_is_ and Δ*G* ^o^ values of the various aliphatic ligand series appear to be primarily associated with the number of methylene groups, with little to no contribution from the carboxylate tail interactions. The study demonstrates that methylene groups, although generating weak van der Waals forces and hydrophobic interactions, contribute to the binding energy of ligands to *Pa*D2HGDH.

**Figure 5:**
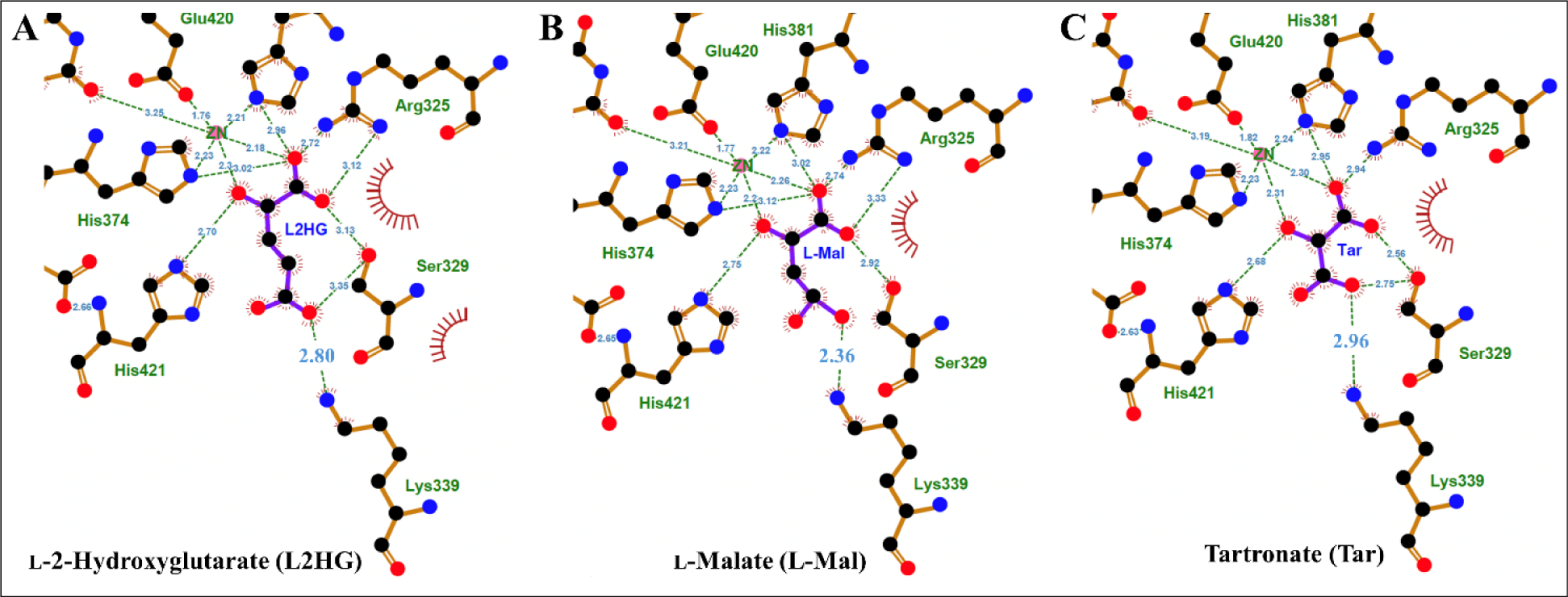
LigPlot+ ^34,35^ ligand contact maps showing aliphatic C2-hydroxyl inhibitor interactions with *Pa*D2HGDH residue K339. A view of the active site interactions of *Pa*D2HGDH with (A) L2HG, (B) l-malate, and (C) tartronate. The *Pa*D2HGDH target residues are shown with tan bonds, ligands with purple bonds, carbon atoms as black spheres, nitrogen atoms as blue spheres, and oxygen atoms as red spheres. Hydrophobic contacts are shown as crimson arches. Hydrogen bonds are shown as green dashed lines. The docking files were generated using the Protenix online tool.^29^

### *Pa*D2HGDH Favors the Binding of Polar Ligands to Nonpolar Counterparts

This conclusion is supported by inhibition studies with various ligands (Table 1). When the aromatic mandelate series was tested as inhibitors, the inhibition constants decreased with an increasing number of hydroxyl substituents on the aromatic ring of mandelate. Additionally, when lactate and tartronate, having a nonpolar tail and a polar tail, respectively, were tested, a lower inhibition constant was recorded for tartronate. The data are consistent with *Pa*D2HGDH favoring the binding of ligands with more polar moieties. This observation is expected, considering the highly polar nature of the *Pa*D2HGDH active site. Due to the enhanced active site interactions of the polar ligands, there is increased binding energy for the ligands, which is observed as a decrease in the ligand inhibition constants for binding the enzyme and, subsequently, the generation of better inhibitors. Thus, increasing polar residue interactions in *Pa*D2HGDH favors the binding of the ligand to the enzyme active site, making polar ligands better inhibitors of *Pa*D2HGDH with relatively smaller *K*_is_ values.

In conclusion, this study has investigated the structural moieties required for ligand recognition to yield various effector outcomes in *Pa*D2HGDH. The study demonstrates that the stereochemistry and functional group at the C2 position of various ligands are the main determinants for ligand outcomes in *Pa*D2HGDH. The enzyme strictly recognizes d-isomeric ligands as substrates, with their l-isomers acting as reversible inhibitors of the enzyme. Ligand binding in *Pa*D2HGDH requires bidentate coordination with the active site Zn^2+^ cofactor, making d-isomeric amino acids that have a positive charge at pH 7.4 neither substrates nor inhibitors of the enzyme. Additionally, hydrophobic and van der Waals interactions of methylene groups contribute small but significant energies to ligand binding. Thus, longer chain ligands, as well as polar ones, have lower *K*_is_ and Δ*G* ^o^ values due to their enhanced interactions with the active site K339 residue and the highly polar active site of *Pa*D2HGDH, respectively, compared to their longer and nonpolar counterparts. The study demonstrates that *Pa*D2HGDH can be reversibly inhibited and serves as a foundation for biochemical studies focused on developing inhibitors targeting *Pa*D2HGDH. While these findings can be directly applied to D2HG biosensor development, efforts targeting *Pa*D2HGDH for therapeutics against *P. aeruginosa* should strive to achieve irreversible inhibition of the enzyme.

## Data Availability

All data are contained within the manuscript.

## Abbreviations

*Pa*D2HGDH: *Pseudomonas aeruginosa* d-2-hydroxyglutarate dehydrogenase
D2HG: d-2-hydroxyglutarate
